# Multi-modal LSTM network for anomaly prediction in piston engine aircraft

**DOI:** 10.1016/j.heliyon.2024.e25120

**Published:** 2024-01-24

**Authors:** Waqas Rauf Khattak, Ahmad Salman, Salman Ghafoor, Seemab Latif

**Affiliations:** School of Electrical Engineering and Computer Science, National University of Sciences and Technology (NUST), Sector H-12, Islamabad, 44000, ICT, Pakistan

**Keywords:** Aircraft piston engine flameout, Predictive maintenance, Machine learning, Deep learning

## Abstract

An aircraft is a highly intricate system that features numerous subsystems, assemblies, and individual components for which regular maintenance is inevitable. The operational efficiency of an aircraft can be maximised, and its maintenance needs can be reduced using an effective yet automatic AI-based health monitoring systems which are more efficient as compared to designing and constructing expensive and harder to operate engine testbeds. It has been observed that aircraft engine anomalies such as undergoing flameouts can occur due to the rapid change in the temperature of the engine. Engine oil temperature and cylinder head temperature, two measures connected to this issue, might be affected differently depending on flight modes and operational conditions which in turn hamper AI-based algorithms to yield accurate prediction on engine failures. In general, previous studies lack comprehensive analysis on anomaly prediction in piston engine aircraft using modern machine learning solutions. Furthermore, abrupt variation in aircraft sensors' data and noise result in either overfitting or unfavourable performance by such techniques. This work aims at studying conventional machine learning and deep learning models to foretell the possibility of engine flameout using engine oil and cylinder head temperatures of a widely used Textron Lycoming IO-540 six-cylinder piston engine. This is achieved through pre-processing the data extracted from the aircraft's real-time flight data recorder followed by prediction using specially designed multi-modal regularised Long Short-Term Memory network to enhance generalisation and avoid overfitting on highly variable data. The proposed architecture yields improved results with root mean square error of 0.55 and 3.20 on cylinder head and engine oil temperatures respectively averaged over three case studies of five different flights. These scores are significantly better i.e., up to 84% as compared to other popular machine learning predictive approaches including Random Forest, Decision Tree Regression, Artificial Neural Networks and vanilla Long Short-Term Memory networks. Through performance evaluation, it can be established that the proposed system is capable of predicting engine flameout 2 minutes ahead and is suitable for integration with the software system of aircraft's engine control unit.

## Highlights

• An automatic AI health monitoring system maximises operational efficiency, reducing the need for expensive engine testbeds.
• Rapid temperature changes in aircraft engines pose challenges for accurate prediction, affecting AI algorithms' performance.
• This study employs multi-modal regularised LSTM network to address the challenge of overfitting on noisy sensor data.
• The proposed architecture achieves up to an 84% improvement in predicting engine flameout vs. state-of-the-art methods.

## 1 Introduction

Aircraft maintenance, fuel costs, increase in air traffic, competition, recession, air pollution, security, and operational difficulties are some of the challenges that the aviation industry continues to face despite its rapid expansion [1]. Aircraft maintenance accounts for approximately 40% of the total operating costs of aviation systems. Having the right health monitoring in place for a given flying service will enhance operational efficiency, while decreasing the need for aircraft repair [2].

Aircraft engines must operate within specific physical constraints due to their costly maintenance [3]. Although modern engines are equipped with more control variables and sensors, they remain susceptible to failures beyond regular wear and tear [4], [5]. Detecting minor issues early can prevent them from escalating into major problems and potentially avoid accidents [6]. The frequency of technical difficulties and accidents is directly linked to the rate of aircraft manufacturing.

The Engine Test Bed (ETB) [7] simulates actual flights for real-time estimation and optimisation of flight characteristics. Flight parameter optimisation systems consist of various Line Replacement Units (LRUs) or equipment [8]. In modern commercial aircraft, extensive data is continuously recorded, encompassing weather conditions, pilot preferences, and system health parameters [9]. Data mining methods are applied to these vast datasets to discover valuable patterns. The primary goal of evaluating such large datasets is to enhance the aircraft's overall health and reduce airline operating costs. Alarm systems in planes are capable of promptly detecting life-threatening issues [10].

Aircraft health parameters and their fluctuations, measured through sensor readings, are gaining attention for improved estimation systems. Effective optimisation of engine parameters can lead to reduced life cycle expenses, engine overhaul costs, and enhanced engine safety [11], [12]. Conventional aviation engine optimisation involves using an ETB facility to analyse and improve the engine's performance in flight [13]. However, ETB is a laborious solution that can only estimate and optimise aircraft engines on the ground, despite their operational differences in the air. Maintenance procedures in the current literature are typically categorised as follows,

• Run-to-failure (R2F) maintenance: A simplest yet expensive maintenance approach, this is after-breakdown maintenance and repair procedure which usually results in high cost of addressing the issue after engine component(s) failure.
• Preventive maintenance (PvM): Preventative maintenance, often known as scheduled maintenance prevents failures but may lead to unnecessary maintenance exercise.
• Predictive maintenance (PdM): In this approach, maintenance is performed as needed, and prediction methods are utilised in planning and scheduling systems to determine the appropriate timing for actions. PdM systems employ ad-hoc or statistical inference-derived health factors.

Usually, statistical, AI, or model-based systems are used to detect and predict machinery health. Model-based methods require mechanical and theoretical knowledge of the equipment, while statistical approaches rely on mathematical backgrounds, leading to the increasing use of AI in PdM applications [14]. AI outperforms statistical methods in predicting equipment failure due to its ability to learn patterns from data and conduct predictive analysis [15]. Prediction algorithms based on machine learning and deep learning are capable of detecting latent data correlations and managing high-dimensional and multivariate data in complex, dynamic scenarios [16]. This study explores how can these algorithms be effectively utilised to predict engine flameout in piston engine aircraft, specifically using engine oil and cylinder head temperatures from a Textron Lycoming IO-540 six-cylinder piston engine widely used in small private and trainer aircraft? Moreover, a thorough empirical assessment of contemporary machine learning and deep learning methods becomes essential to validate their applicability, particularly when dealing with limited data and substantial variations in flight patterns and, consequently, aircraft sensor readings in real-world scenarios. The contributions of this study are summarised below.

• Arrange mission data pertaining to various flight phases after organising and extracting from an aircraft's flight data recorder (FDR).
• Perform a thorough pre-processing of the FDR data for cylinder head temperature and engine oil temperature.
• Analyse popular machine learning prediction approaches including Random Forests (RF), Decision Tree Regression (DTR) and Artificial Neural Networks (ANN).
• Proposing a stacked Long Short-Term Memory (LSTM) network with regularised multi-modal input scheme for accurate prediction and mitigating the challenge of unreliable performance on our data exhibiting high variation.

## 2 Related work

This section summarises research, especially related to machine learning and deep learning on estimating an aircraft engine's health metrics including remaining usable life (RUL), a measure which can be used to estimate ideal time for maintenance or service [17]. Mostly, the literature discusses turbojets, turbofans, turboprops, and turbo-shafts engines whereas piston engines, although widely used, are relatively less studied. The features mostly used to predict RUL and flameouts include Exhaust Gas Temperature (EGT), Cylinder Head Temperature (CHT), Engine Oil Temperature (EOT), Low Pressure Compressor (LPC), High Pressure Compressor (HPC), Low Pressure Turbine (LPT), High Pressure Turbine (HPT), N1 and N2 speed, Angle of Attack, Engine RPMs, Pitch, Roll, and Vertical Acceleration. These features are acquired using various sensors mounted in aircraft's electronic and control systems.

The prediction of RUL and flameouts due to anomalous behaviour in sensors' data is helpful in predictive maintenance of aircraft engines. In pursue of data-driven approaches, ensemble regression approach is proposed in [18] to estimate RUL where random forest and gradient boosting regression models are trained using FEMTO ball bearings data (IEEE PHM Data Challenge 2012). Likewise, in [19], a double-CNN architecture for accurate RUL prediction is presented which brings significant improvements to the prediction reliability. The recommended framework relies on feature extraction to preserve and utilise information regarding aircraft's engine health. LSTM and Gated Recurrent Units (GRU)-based Recurrent Neural Networks (RNN) are used in [20] for estimating the approximate service period of a turbofan engine with over 90% accuracy on an engine degradation simulation dataset. A similar outcome is presented for PdM using LSTM and GRU in [21] and bidirectional LSTM in [22]. The utilisation of RNN in aircraft RUL estimation is not new though as previous attempts are successful in proving the prediction capabilities of RNNs [23]. RF regression is employed to predict aircraft component failure as PdM in [24], [25] by collecting data from various engine sensors. Similarly, a combination of CNN and LSTM is used in [26] to predict RUL using a post flight data achieving an accuracy of 99% on a selected flight dataset. In [27] 1D CNN with Monte Carlo dropout is used in a reinforcement learning manner for estimating turbofan engine RUL with impressive success rate and dropping maintenance cost by 29.3% validating the similar previous study [28], [29] which utilise related deep CNNs.

Turbojet engines are also studied in detail for PdM as [26], [30], [31] employ LSTM and Multiple Instance Regression (MIR) on engine degradation and post-flight datasets with promising results. Similarly, LSTM with Support Vector Machines (SVM) presents a hybrid model for RUL prediction on NASA's aero-engine dataset [32]. On the other hand, [33] proposes RUL estimation using multiple machine learning modules including SVM, K-Nearest Neighbour (KNN), RF, and Analysis of Variance (ANOVA) statistical approaches. In [34] a combination of 1D CNN, autoencoder, and bidirectional GRU network is used on time-series data of 60 turbojet commercial aircraft in a pursue of detecting rare engine failures. This approach primarily addresses data imbalance to achieve favourable performance. DTR and ANN are among the popular approaches used in PdM for RUL in gas turbine engines as demonstrated by [35], [36] where a simulated dataset is used to prove the proposed algorithm's efficacy. In these studies of gas turbine engines, LPC, HPC, LPT, and HPT are the most commonly used features along with a few others for better prediction.

The comprehensive literature review found that on average only 3.7 articles per year appear to address PdM of aircraft engines using machine learning and deep learning. Furthermore, as mentioned earlier, piston engines are relatively less studied in the pursue of PdM and due to their simpler structural arrangement, challenges like flameout occur more frequently. Therefore, this work aims to target piston engines with CHT and EOT as features for predicting such anomalies. As the Textron Lycoming IO-540 engine is used in our study, the aircraft that come with this engine e.g., Cessna 188, 206 and several other trainer aircraft lack ejection seat system making it imperative for the pilot to initiate landing protocol or corrective measures in case of engine flameout by varying air-fuel mixture ratio, throttle and primer. In terms of PdM, the CHT and EOT long-term data can be used to detect malfunctioned engine components for replacement hence playing critical role in estimating engine's RUL.

In the context of various studies discussed earlier, utilising either traditional machine learning or contemporary deep architectures, a research gap emerges with two crucial aspects. Firstly, cutting-edge deep learning techniques such as CNN, LSTM, and GRU networks, characterised by intricate and highly nonlinear mathematical structures, sometimes struggle to demonstrate robust generalisation capabilities when confronted with related yet diverse and small data exhibiting high variability [37]. Despite achieving favourable outcomes on specific datasets, their performance on data representing different flight patterns often compromises anomaly prediction accuracy due to overfitting and a lack of data-specific regularisation constraints. These architectures usually apply data augmentation approached to extend data samples and avoid overfitting. However, aircraft data involving various sensors and natural flight patterns should not be augmented with random transformations to the original data as doing so may undermine the actual sensor behaviour leading to an anomaly. On the other hand, commonly employed traditional machine learning approaches like DTR, RF, and ANNs typically face challenges in extracting complex sequential features from data, hindering their ability to effectively learn task-specific information for aircraft anomaly prediction, particularly beyond a certain time span [38]. The proposed scheme focuses on resolving these critical issues, aiming to attain high accuracy in predicting piston engine flameout within a small yet highly variable dataset of real flights. This is accomplished by extracting meaningful patterns from the data through a residual LSTM setup, complemented by data signal pre-processing for effective regularisation to avoid overfitting.

## 3 Materials and methods

This section addresses the whole pipeline comprising data collection, data pre-processing, and the proposed deep architecture for engine flameout prediction. As a result, the analytical study is based on full flights as well as cruise phases to study various prediction algorithms of machine learning and deep learning. In this regard, the proposed regularised multi-modal LSTM model is compared with popular DTR, RF, standard LSTM and ANN. The nomenclature of symbols used in the equations for explaining data pre-processing and the proposed architecture is given in Table 1.

**Table 1:** Nomenclature of Symbols.

**Latin Symbols**	
*b*	Bias vector of Long Short-Term Memory layer
*C*	Cell state of Long Short-Term Memory network
*F*	Forget gate of Long Short-Term Memory network cell
*H*	Hidden state of Long Short-Term Memory network cell
*L*	Total number of layers in Long Short-Term Memory network
*n*	Number of data samples
*O*	Output gate of Long Short-Term Memory network cell
*r*	Correlation coefficient
*s*	Standard deviation
*t*	Time
*T*	Length of input and output sequences of the proposed network
*U*	Recurrent weight matrix of Long Short-Term Memory network
*W*	Weight matrix of Long Short-Term Memory network
*x*	Independent data variable in correlation measure
*X*	Input sequence to Long Short-Term Memory network
*y*	Dependent data variable in correlation measure
*Y*	Network output of Long Short-Term Memory network
*z*	Data sample normalised with mean and standard deviation

**Greek Symbols**	

*α*	First layer of residual/skip connection
*β*	Second layer of residual/skip connection
*μ*	Mean
*σ*	Sigmoid activation function in Long Short-Term Memory network

**Subscripts**	

_*k*_	Long Short-Term Memory Network branch number

**Superscripts**	

^*α*^	Same as *α*
^*β*^	Same as *β*
^*i*^	Denotes input gate of Long Short-Term Memory network cell
^*f*^	Denotes forget gate of Long Short-Term Memory network cell
^*c*^	Denotes cell state of Long Short-Term Memory network
^*o*^	Denotes output gate of Long Short-Term Memory network cell
^*l*^	Denotes layer number of Long Short-Term Memory network
^*L*^	Denotes last layer of Long Short-Term Memory network
^*y*^	Denotes Long Short-Term Memory Network output

**Operational Symbols**	

⊙	Element-wise multiplication of vectors/matrices
⌢	Concatenation of vectors/matrices

### 3.1 Dataset and pre-processing

The dataset used in experimentation is collected from FDR of an aircraft housing Textron Lycoming IO-540 six-cylinder piston engine. The data is acquired in a customised protocol of Garmin_™_ as their equipment is used. With the help of GPS altitude, the flight phases with vital sensory data are extracted, while removing all the other unnecessary parameters of the mission profiles. Consequently, overall dataset comprises 11 flights out of which 5 flights are kept for training different algorithms, 5 are kept for testing, and remaining one for validation of optimum parametric settings in training those algorithms. The duration of flights ranges from 30 to 65 minutes. The flights reserved for training come without any flameout anomaly therefore, we call them healthy flights. In contrast, flights in test dataset experience flameouts due to EOT and CHT exceeding their flameout threshold of $210^{o}F$ and $450^{o}F$ respectively for the engine type under study.

**Figure 1:**
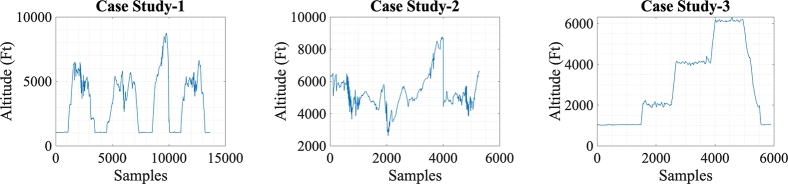
Case Studies: From left to right, concatenation of four complete flights (Case Study-1), cruise phases (Case Study-2) and one complete multi-altitude cruise flight (Case Study-3). Altitude is given in mean sea level (MSL).

After extracting and visualising FDR data, three case studies are presented to train all the algorithms including the proposed. For Case Study-1 all flights are combined to form one long duration flight. In other words, the flight data is generated by concatenating 4 out of 5 flights reserved for training to generate a bigger training dataset. For Case Study-2 cruise phases from these 4 flights are isolated and then combined and for Case Study-3 data, the fifth standalone flight showing a complete mission profile with multiple cruise phases at different altitudes is used. This data segregation of flight phases is achieved using GPS altitude monitoring. All the three case studies are shown in Fig. 1. The case-wise study helps us in judging the generalisation potential of learning algorithms to favourably perform on test dataset when trained on three different scenarios including flights with takeoff and landing phases and flights with cruise-only phases. It is worth mentioning that test dataset comes naturally without data segregation except the case when models are trained in Case Study-2, the test data of all five flights also include only cruise phases, the reason of which is explained in Results and Discussion section.

It is important to note that the data from FDR is recorded at 1 Hz sampling frequency i.e., the samples in Fig. 1 corresponds to seconds in time. Since features like control surface motion, temperature, vibration, and combat sensor data are highly variable and dependent on flight trend, predicting an engine flameout is crucial. To improve model performance, selecting appropriate attributes to describe various flight stages is important as incorporating irrelevant variables into the training process can harm model performance. Feature selection enhances predictor performance, reduces overfitting and redundant data, shortens training time, and improves model quality. To achieve this, correlation method is used for feature selection as independent and dependent features have a linear relationship. This work focuses on multivariate regression and uses the correlation technique using (1) to select attributes for the different flight phases. EOT and CHT are the dependent variables in (1) presented as *y*, while the independent features are represented by *x*. We have chosen 15 most relevant features out of the sample size *n* of 31, including highly correlated features such as EOT and CHT. Relevant feature selection, as the final step in data preparation, helps eliminating noise and unnecessary data which supports better training.

(1) $$r=\frac{n\left(\sum xy\right)-\left(\sum x\right)\left(\sum y\right)}{\sqrt{\left[\left(n\sum x^{2}\right)-{\left(\sum x\right)}^{2}\right]\left[\left(n\sum y^{2}\right)-{\left(\sum y\right)}^{2}\right]}}$$

**Figure 2:**
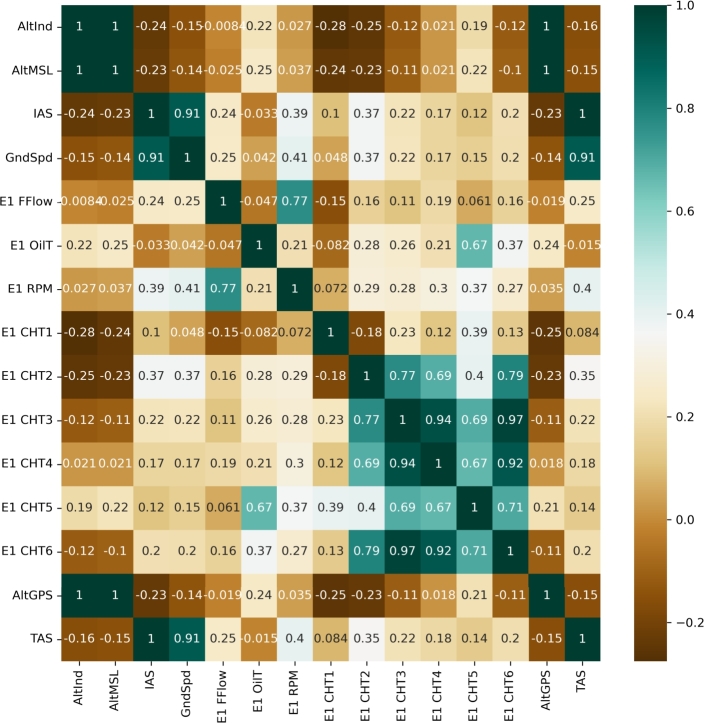
Correlation heatmap for 15 features.

In each of the three case studies, a selection of 15 features is made to represent the outputs of various sensors on the aircraft. These features include Cylinder Head Temperatures (CHT) for all six cylinders, True Air Speed (TAS), Indicated Air Speed (IAS), Ground Speed (GndSpd), Revolution Per Minute (RPM), Engine Fuel Flow (Fflow), Altitude GPS (ALtGPS), Altitude Mean Sea Level (ALTMSL), Altitude Indicated (ALTInd), and Engine Oil Temperature (EOT). These features are chosen as they exhibit dependency with each other. The combined data of all case studies are analysed and a correlation heat map is generated which indicates direct or indirect relation among features as shown in Fig. 2. These 15 features in the form of time-series data are employed for training all algorithms to detect engine flameout. In case of standard and the proposed multi-modal LSTM network, all those 15 features' time-series data are taken as separate input channels (analogous to CNN's input channels). The LSTM layers then process each channel independently, allowing the network to capture the relationships and patterns among features that may help predict behaviour of CHT and EOT at the output. From Fig. 2 it is evident that EOT shows high correlation with CHT5, which in turns is directly related to other five CHTs. For this study, the temperature of the sixth cylinder (CHT6) and engine oil temperature give enough information to predict flameout. After feature selection, scaling, normalisation, and standardisation of data *x* is done as pre-processing step using (2) and (3) to get the normalised data $x_{norm}$. Equation (3) limits the data in the range [0, 1]. This pre-processing assumes data to be Gaussian distributed. [39]. To sum up, for Case Study-1, all the waveforms of 15 features are concatenated separately (to make 15 long waveforms) with their values representing take-off, cruise and landing phases of four different flights. Similarly, in Case Study-2, only feature values of cruise phases of these four flights are concatenated, making 15 different waveforms of cruise phases, while Case Study-3 is a single long flight with take-off, landing and multiple cruise phases making 15 different waveforms for this single flight.

(2) $$z=\frac{\left(x-u\right)}{s}$$

(3) $$x_{\text{norm }}=\frac{z-\min(z)}{\max(z)-\min(z)}$$

### 3.2 Deep architecture

**Figure 3:**
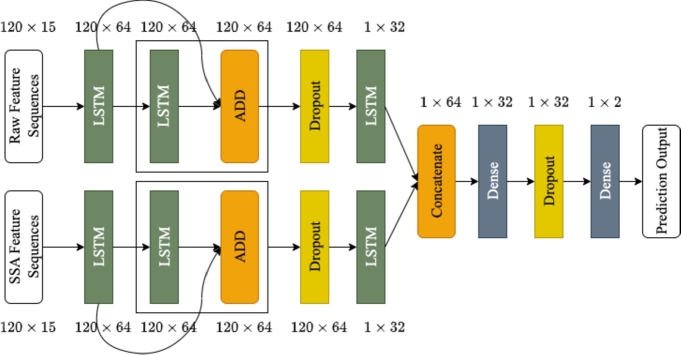
The proposed multi-modal deep architecture.

Here, a deep multi-modal regularised LSTM network is presented for engine flameout prediction using waveforms of data features extracted from FDR. As illustrated in Fig. 3, the proposed network is based on LSTM layers arranged in two branches to extract waveform patterns from two separate input representations i.e., raw time-series waveforms of data features and their smoothed versions after applying Singular Spectrum Analysis (SSA) [40]. The SSA-applied waveforms as inputs to the deep architecture act as regularisation constraint to alleviate the impulsive and noise-like behaviour of time-series data. This in turn supports the prediction capacity of LSTM architecture by avoiding overfitting on the raw signal fluctuation. As oppose to other approaches like moving-average or exponential smoothing, SSA is a data-driven method that does not rely on preset assumptions or parameters. It extracts the underlying patterns and components directly from the data without imposing any specific mathematical model. This makes SSA more flexible and adaptable to different types of time series. In the proposed network, both branches comprise three LSTM layers each, where a residual LSTM block of two layers precedes the third layer. Outputs of the last LSTM layers from both branches are combined and passed through the dense layers to achieve predicted output.

Assuming input and output sequences for the proposed network for each data feature to be $X_{k}=[X(1),X(2),\ldots ,X(T)]$ and Y=[Y(1),Y(2)] respectively. For k=1,2, the input $X_{1}$ is raw feature time-series data, while $X_{2}$ is its SSA counterpart yielding *Y* as output of the network predicting samples of EOT and CHT6 waveforms. Using weight matrices $W_{k}^{i}$, $W_{k}^{f}$, $W_{k}^{c}$, $W_{k}^{o}$, recurrent weight matrices $U_{k}^{i}$, $U_{k}^{f}$, $U_{k}^{c}$, $U_{k}^{o}$, and bias vectors $b_{k}^{i}$, $b_{k}^{f}$, $b_{k}^{c}$, $b_{k}^{o}$, the inference of the proposed network for layer l=1,…,L and branch *k* is given in (4)-(12),

Input gate at time *t*:

(4) $$I_{k}^{\left(l\right)}(t)=\sigma (W_{k}^{i(l)}X_{k}(t)+U_{k}^{i(l)}H_{k}^{\left(l-1\right)}(t)+b_{k}^{i(l)})$$

Forget gate at time *t*:

(5) $$F_{k}^{\left(l\right)}(t)=\sigma (W_{k}^{f(l)}X(t)+U_{k}^{f(l)}H^{{\left(l-1\right)}_{k}}(t)+b_{k}^{f(l)})$$

Cell state at time *t*:

(6) $$C_{k}^{\left(l\right)}(t)=F_{k}^{\left(l\right)}(t)⊙C_{k}^{\left(l\right)}(t-1)+I_{k}^{\left(l\right)}(t)⊙\tanh(W_{k}^{c(l)}X(t)+U_{k}^{c(l)}H_{k}^{\left(l-1\right)}(t)+b_{k}^{c(l)})$$

Output gate at time *t*:

(7) $$O_{k}^{\left(l\right)}(t)=\sigma (W_{k}^{o(l)}X(t)+U_{k}^{o(l)}H_{k}^{\left(l-1\right)}(t)+b_{k}^{o(l)})$$

Hidden state at time *t*:

(8) $$H_{k}^{\left(l\right)}(t)=O_{k}^{\left(l\right)}(t)⊙\tanh(C_{k}^{\left(l\right)}(t))$$

In the proposed network, the inputs to the last LSTM layers (l=L) of each branch are

(9) $$Hi_{k}^{\left(L\right)}(t)=H_{k}^{\left(L-\alpha \right)}(t)+H_{k}^{\left(L-\beta \right)}(t)$$

where, $Hi_{k}^{\left(L\right)}(t)$ represent hidden states of residual blocks. Now, for two branches (k=1,2) of the proposed network,

(10) $$H_{1}^{\left(L\right)}(t)=O_{1}^{\left(L\right)}(t)⊙\tanh(C_{1}^{\left(L\right)}(t))$$

(11) $$H_{2}^{\left(L\right)}(t)=O_{2}^{\left(L\right)}(t)⊙\tanh(C_{2}^{\left(L\right)}(t))$$

The network output is

(12) $$Y(t)=H_{1}^{\left(L\right)}{\left(t\right)}^{⌢}H_{2}^{\left(L\right)}(t)$$

In practice, L=4, α=1 and β=2 for experimentation reported here. To simplify, mathematical expressions for dropout and dense layers are dropped (see Fig. 3).

## 4 Results and discussion

**Figure 4:**
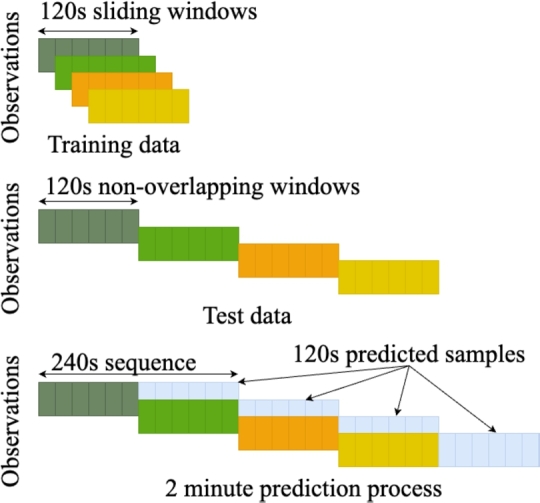
Illustration of training and test data arrangement.

**Figure 5:**
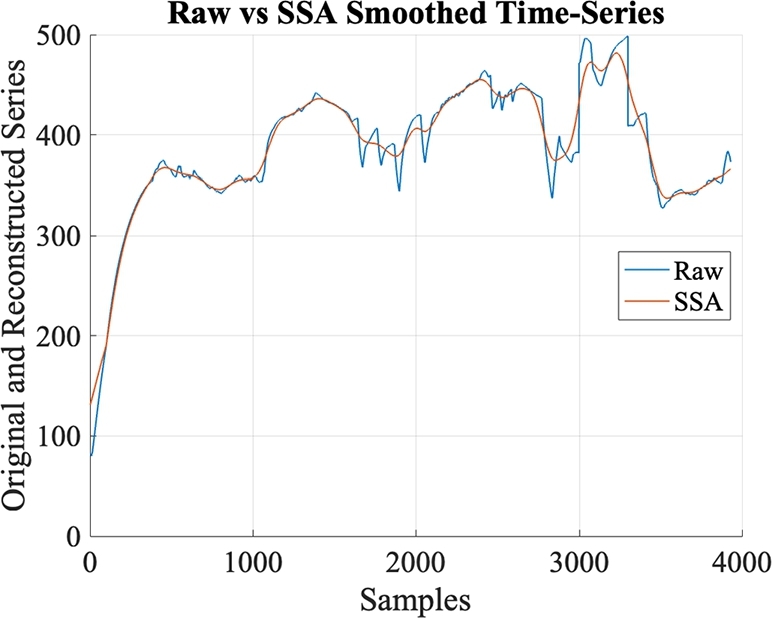
Illustration of raw time-series and its smoothed version using SSA.

The proposed architecture takes time-series training data comprising 15 features as input arranged as 120 s sliding time-window. In other words, a group of 120 s time series sequences are processed at the input layer at a time and each next sequence is 1 s shifted in a sliding window as shown in Fig. 4. The raw time series data and its smoothed SSA version for one of 15 features is shown in Fig. 5 for illustration.

As previously mentioned, the training data is organised into three case studies to carefully evaluate the performance of prediction algorithms, including the proposed one. Each algorithm is trained independently using the training data specific to each case study. For Case Study-1, the training dataset consists of the concatenation of data from four flights to form a larger dataset of 13,657×120×15 dimension (observations × time-steps × feature dimension). Similarly, the data dimensions are 5,396×120×15 and 5,907×120×15 for Case Study-2 and Case Study-3 respectively. For training data, as illustrated in Fig. 4, observations are extracted using sliding windows of 120 s (two minutes) duration while test data is arranged using non-overlapping windows. The prediction on test data is made in a special manner to estimate CHT6 and EOT values 120 s ahead. To achieve this, a test data observation of 120 s length (120 samples) is applied as input to the prediction algorithms including the proposed one and sample value at 121 s is predicted. In the next iteration, the predicted sample is included in the input sequence, while the first sample is excluded. This sliding window prediction operation is continued till $240^{th}$ sample for each observation in test data (shown in blue in Fig. 4) and must be completed before the arrival of the first sample of next observation. The flavours of algorithms including ANN, DTR, and RF are taken from [36] and [33]. The ANN is a 7-layer fully connected neural network with 20, 15, 10, 8, 5, 4, and 3 neurons in the successive hidden layers, 15 dimensional input, and 2 outputs for EOT and CHT6 regression. The layers use sigmoid activation and minimum mean square error (MSE) as loss function to train the ANN for 1,000 epochs for each case study. In the case of DTR, a random state of 0 is employed, whereas for RF, 100 estimators are utilised, and a random state of 0 is used during training for each case study. The proposed multi-modal LSTM network is trained with batch size of 32 and 50 epochs with learning rate of 0.001. For SSA smoothing, we use 1^st^ component to generate waveforms. The training and testing is performed on Intel® Core™i7, 32 GB RAM and Nvidia® Titan X GPU with TensorFlow.

**Figure 6:**
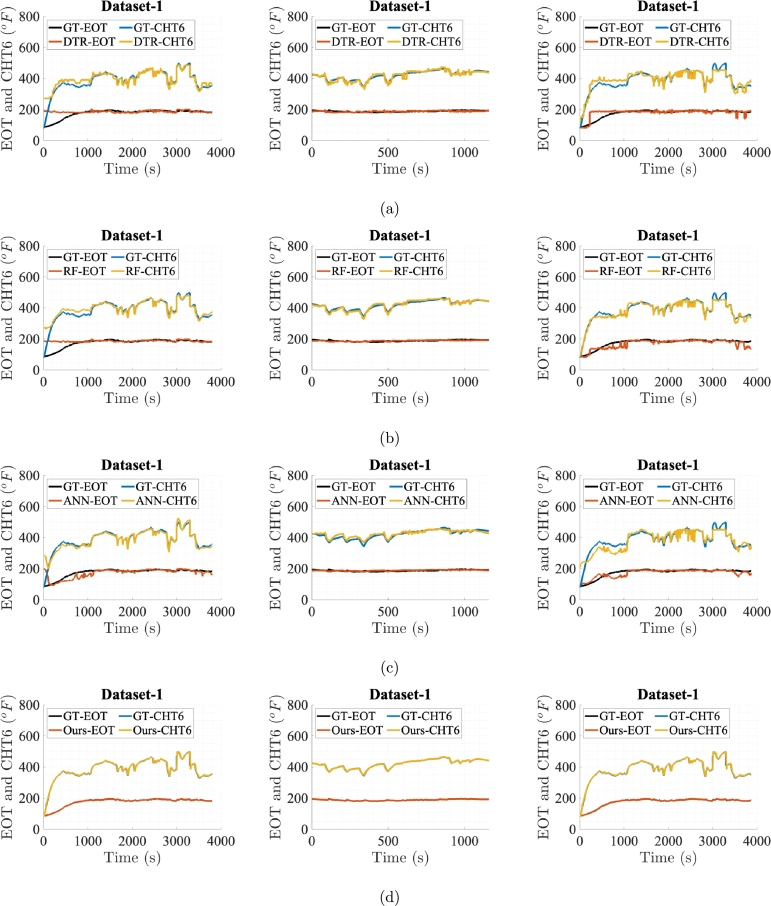
Prediction results on test Dataset-1 with (a), (b), (c), and (d) represent outcomes of DTR, RF, ANN, and the proposed method respectively for the three case studies each.

**Figure 7:**
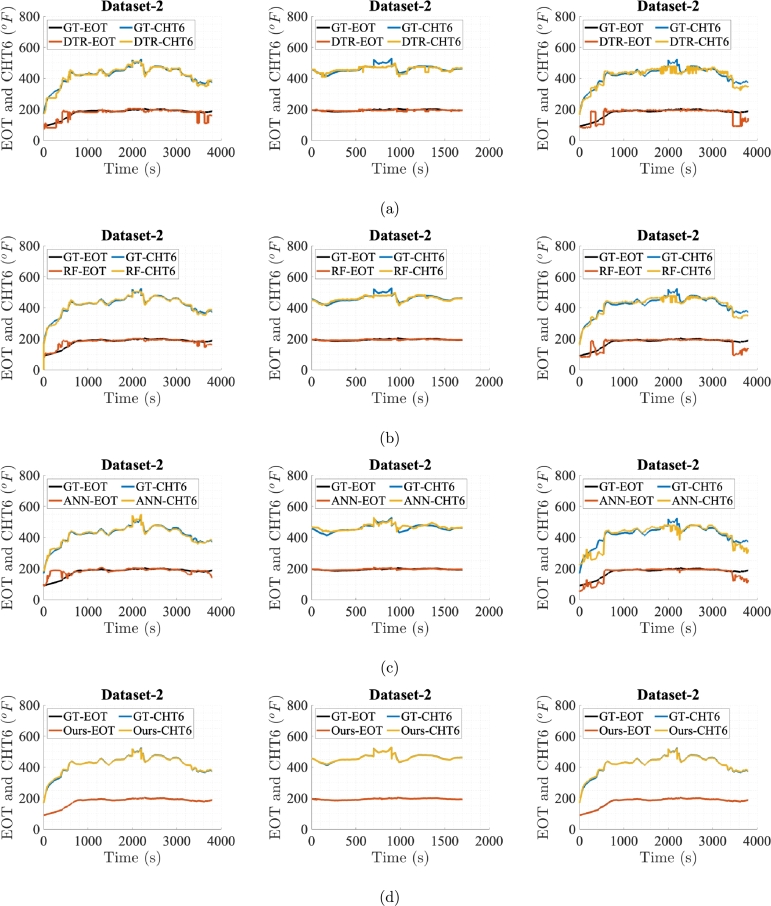
Prediction results on test Dataset-2 with (a), (b), (c), and (d) represent outcomes of DTR, RF, ANN, and the proposed method respectively for the three case studies each.

**Figure 8:**
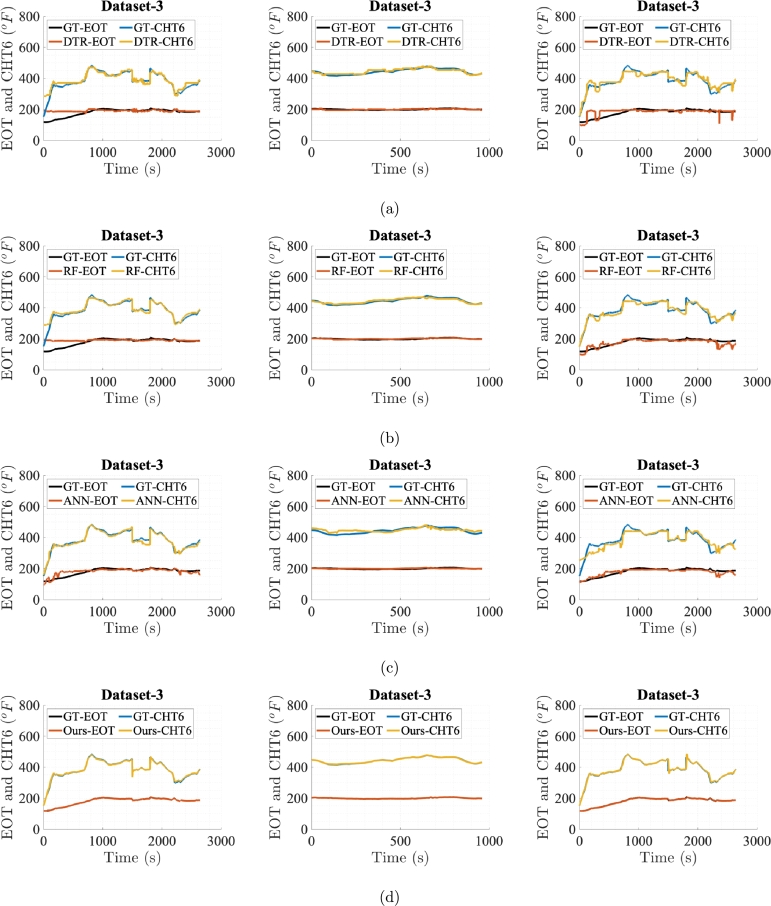
Prediction results on test Dataset-3 with (a), (b), (c), and (d) represent outcomes of DTR, RF, ANN, and the proposed method respectively for the three case studies each.

**Figure 9:**
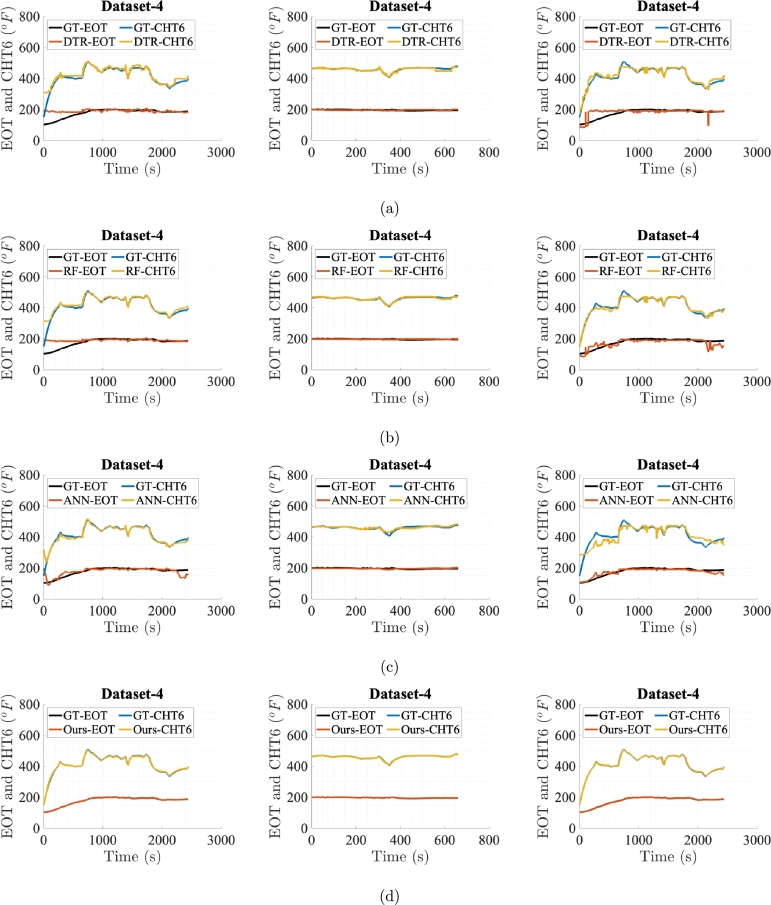
Prediction results on test Dataset-4 with (a), (b), (c), and (d) represent outcomes of DTR, RF, ANN, and the proposed method respectively for the three case studies each.

**Figure 10:**
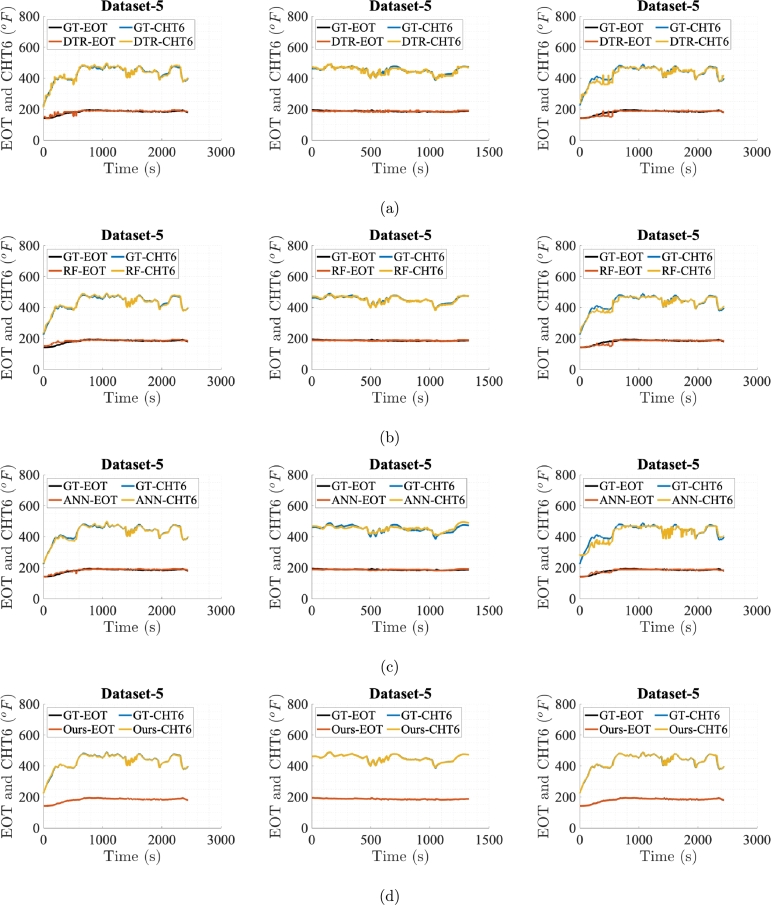
Prediction results on test Dataset-5 with (a), (b), (c), and (d) represent outcomes of DTR, RF, ANN, and the proposed method respectively for the three case studies each.

Figure 6, Figure 7, Figure 8, Figure 9, Figure 10 show prediction performance of ANN, DTR, RF, and the proposed algorithm for all three case studies on test dataset. The five flights in the test dataset are dubbed as test Dataset-1 to Dataset-5. To visually demonstrate performance and highlight the accuracy of direct outputs, the prediction curves for CHT6 and EOT produced by each algorithm are juxtaposed with their respective ground truth (GT) curves. These figures indicate that accurate predictions should align with corresponding samples on the ground truth curve, leading to enhanced prediction accuracy. Conversely, inaccurate predictions result in discrepancies between the predicted and ground truth samples. Therefore, visual comparing of the predicted and ground truth curves, provides a tangible representation of how well the algorithm captures and predicts the observed patterns in the data. The degree of alignment between the predicted and ground truth curves serves as a visual indicator of the algorithm's accuracy in forecasting sequences, showcasing its effectiveness in capturing the underlying patterns inherent in the dataset. It is evident that the proposed algorithm, in general, meticulously follows the trend of ground truths for both EOT and CHT6 curves in prediction. For Case Study-1, ANN, DTR, RF show better results in predicting CHT6 but show faulty trends for EOT with higher error. In Case Study-1 and 3, ANN, DTR, and RF show larger deviation in following both CHT6 and EOT envelops when compared to the ground truths. Also the noisy and jittery behaviour of these algorithms especially by DTR and RF shows inability of these approaches to generalise and extract natural pattern from the training sequences which results in impulsive behaviour on test data. In contrast, the proposed algorithm takes advantage of SSA regularisation and learns the pattern of CHT6 and EOT behaviour based on information extraction from the input feature space on training data which eventually results in relatively accurate sequence envelop of both CHT6 and EOT.

Apart from prediction curves, Table 2 tabulate the average performances in terms of root mean square error (RMSE), mean absolute error (MAE) and r-squared ($r^{2}$) for all five test flights for the three case studies. These metrics quantify how well a regression model fits a dataset for prediction. In a separate experiment, various empirical evaluations on standard LSTM network without SSA branch are performed. This system results in a little overfitting on the training data and fails to yield the prediction outcome with high accuracy on test data. The results are generally inferior to the proposed regularised multi-modal LSTM network but better than DTR, RF, and ANN. Typically, LSTM-based recurrent neural networks stand out as state-of-the-art in predicting anomalies in aircraft engines, especially for turbojet and turboprop engines. Surprisingly, these advanced networks have not been applied to piston engine aircraft. This research delves into their potential for predicting flameout in piston engines, serving as the primary motivation for the proposed LSTM architecture.

**Table 2:** Performance evaluation averaged on all five test datasets for three case studies.

**Average scores on five test datasets for all three case studies**							
**Case Study**	**Algorithm**	**CHT6**			**EOT**		
		**RMSE**	**MAE**	**r**^2^	**RMSE**	**MAE**	**r**^2^

**CaseStudy1**	**DTR**	22.02	12.67	0.13	19.34	12.31	0.88
	**RF**	20.66	11.48	0.21	17.21	9.74	0.91
	**ANN**	15.91	9.40	0.58	13.32	7.72	0.94
	**LSTM**	4.11	3.43	0.98	3.87	3.02	0.99
	**Ours**	0.52	0.44	0.99	3.99	2.60	0.99

**CaseStudy2**	**DTR**	4.23	3.51	-0.37	9.42	6.95	0.78
	**RF**	3.40	2.80	0.08	6.65	5.30	0.89
	**ANN**	3.46	2.91	0.02	10.74	8.68	0.70
	**LSTM**	2.31	1.99	0.87	3.17	2.94	0.90
	**Ours**	0.32	0.24	0.99	1.26	0.83	0.99

**CaseStudy3**	**DTR**	20.18	12.25	0.32	18.62	14.44	0.89
	**RF**	16.46	10.65	0.54	15.10	11.41	0.93
	**ANN**	12.39	8.41	0.74	27.22	19.35	0.78
	**LSTM**	4.54	3.55	0.97	6.09	4.66	0.98
	**Ours**	0.83	0.57	0.99	4.36	2.37	0.99

The graphs and evaluation scores indicate that in Case Study-1, ANN, DTR, and RF models, in general, fail to predict the takeoff and landing phases of flights while focusing on predicting the cruise phase. This may be due to the reason that the takeoff and landing phases of aircraft in manual controlling vary for each flight, causing the models to consider these phases as outliers and unable to learn sequence pattern. Since the cruise phase is typically the longest phase of an aircraft's mission and the sensor data during this phase exhibits less variation, the prediction models prioritise predicting this phase. To effectively capture the takeoff and landing phases, a comprehensive training dataset specific to these phases is required. Due to limited data in these evaluations, ANN, DTR, and RF models result in high RMSE and MAE errors and lower $r^{2}$ scores on the test data flights showing inability to yield true sequence trends.

In Case Study-2, for the training data, the cruise phases are extracted from the dataset used in Case Study-1. While testing against models trained on Case Study-2, test data for all 5 flights also comprise only cruise phases. The reason is to clearly see the performances when training and testing is done only on the cruise phases. As expected, compared with the Case Study-1, the graphs and evaluation scores in Case Study-2 show reduction in the RMSE and MAE values for all prediction models with the proposed model surpassing the rest. However, for CHT6, due to very high ratio of RMSE to the variance of ground truth data relative to the same ratio in Case Study-1 and 3, the $r^{2}$ value is very small. In this scenario, the DTR, RF and ANN predictions are more accurate on individual data points, leading to lower RMSE and MAE values. However, the model might not be capturing the overall variability in the data as effectively as in Case Study-1 and 3, resulting in a smaller $r^{2}$ value.

In Case Study-3, a dataset with a single long and a good mission profile is selected for the training. RF and ANN performance in this case study is similar where ANN outperforms RF in predicting CHT6 while lags in the case of EOT. This is due to behaviour of ANN when training data is very limited and network overfits to the dominant CHT6 values which are always higher than EOT. The proposed model's results remain favourable in this case study too.

To sum up, CHT6 and EOT values are predicted two minutes ahead using ANN, DTR, RF, and the proposed system. In a separate experiment not reported here, prediction window size was increased up to five minutes but very sharp deterioration in prediction accuracy by all the algorithms was observed. Although two minutes still pose a challenging situation for a pilot to carry out counter-flameout measures, it is still a reasonable time to avoid flameouts in a simpler piston engine as compared to jet engines. Indeed, pilot's experience and situation awareness play vital role in such scenarios. In another set of experiments not reported here, we cross evaluated the case studies i.e., we took models trained on Case Study-1 and 3 and tested on the test data of Case Study-2, which only comprises cruise phases. The performances of models trained on the larger data of Case Study-1 outperformed the models trained on the data of Case Study-3.

In this investigation, various prediction algorithms are employed, including the proposed one, to anticipate flameout events two minutes in advance using CHT6 and EOT. All the algorithms, including the suggested architecture, exhibit diminishing performance when forecasting beyond the 2-minute mark, and accuracy sharply declines after 5 minutes, rendering these systems impractical for predictive analysis and maintenance. As indicated in the results, the predicted CHT6 and EOT waveforms are integrated into a recursive prediction process, where an initial input of 120 samples is utilised to estimate the subsequent 120 samples. Notably, small prediction errors, when accumulated during the estimation of samples beyond 120 seconds, contribute to an overall deterioration in prediction accuracy.

Upon careful examination, it is determined that, with a 120 s sliding time window sequence, the proposed flameout prediction pipeline achieves inference completion for predicting the next 120 seconds of CHT6 and EOT in 0.21 seconds on the Nvidia® Titan X GPU and 0.59 seconds on Jetson AGX Xavier, respectively. This makes the system well-suited for real-time applications on edge computing platforms and its potential deployment on the aircraft embedded system under the DO-178C standard titled “Software Consideration in Airborne Systems and Equipment Certification”. Additionally, considerations for software criticality levels (DAL A to E) and the qualification of software development tools under DO-330 must be taken into account.

## 5 Conclusion

In this study, a multi-modal regularised LSTM architecture is introduced for the prediction of engine oil temperature and cylinder head temperature in the Textron Lycoming IO-540 six-cylinder piston engine. The forecasting of these features is deemed crucial for the anticipation of anomalies such as engine flameout, providing pilots with the necessary time to manipulate air-fuel mixture, throttle, or primer settings. Additionally, this predictive model is considered a valuable tool for off-air predictive maintenance of aircraft engines. It is demonstrated that predictions achieved through the proposed approach exhibit superior accuracy compared to other widely-used techniques. The algorithm, specially crafted to address the challenge of overfitting prevalent in limited training data scenarios, renders the proposed system suitable for integration with the aircraft's electronic control unit, facilitating real-time analysis for the early detection of potential flameout situations. It is noteworthy that the proposed recurrent network-based algorithm, while effective, is constrained in predicting flameouts only up to 2 minutes in advance. Future plans involve the acquisition of a more extensive dataset and the training of the system to better adapt to varying mission profiles. The next iteration of this work aims to incorporate transformer-based predictive analysis, allowing the model to capture long-term dependencies and extract information, ultimately extending the prediction window beyond 5 minutes.

## CRediT authorship contribution statement

**Waqas Rauf Khattak:** Visualization, Validation, Software, Methodology, Investigation. **Ahmad Salman:** Writing – original draft, Supervision, Software, Methodology, Formal analysis, Conceptualization. **Salman Ghafoor:** Writing – review & editing, Resources. **Seemab Latif:** Writing – review & editing, Resources.

## Declaration of Competing Interest

The authors declare that they have no known competing financial interests or personal relationships that could have appeared to influence the work reported in this paper.

## Data Availability

Data and source code used in this study will be made available on request.
